# Interaction between Pain, Disability, Mechanosensitivity and Cranio-Cervical Angle in Subjects with Cervicogenic Headache: A Cross-Sectional Study

**DOI:** 10.3390/jcm10010159

**Published:** 2021-01-05

**Authors:** Patricia Martínez-Merinero, Fernando Aneiros Tarancón, Javier Montañez-Aguilera, Susana Nuñez-Nagy, Daniel Pecos-Martín, Rubén Fernández-Matías, Alexander Achalandabaso-Ochoa, Samuel Fernández-Carnero, Tomás Gallego-Izquierdo

**Affiliations:** 1 Physiotherapy Department, Faculty of Health, Exercise and Sport, European University, Villaviciosa de Odón, 28660 Madrid, Spain; patricia.m.merinero@gmail.com; 2 Physiotherapy Center, Alcalá University, 28871 Alcalá de Henares, Spain; fernandoaneirost@gmail.com; 3 Department of Physiotherapy, Universidad Cardenal Herrera-CEU, 46115 Alfara del Patriarca, Spain; Francisco.monta@uchceu.es; 4 Department of Physiotherapy and Nursing, Alcalá University, 28871 Alcalá de Henares, Spain; susana.nunez@uah.es (S.N.-N.); daniel.pecos@uah.es (D.P.-M.); samuel.fernandezc@uah.es (S.F.-C.); tomas.gallego@uah.es (T.G.-I.); 5 Research Institute of Physical Therapy and Pain, Alcalá University, 28871 Alcalá de Henares, Spain; ruben.fernanmat@gmail.com; 6 Department of Health Sciences, Universidad de Jaén, 23071 Jaén, Spain

**Keywords:** forward head posture, mechanosensitivity, pressure pain threshold, cervicogenic headache

## Abstract

The relationship between the forward head posture and mechanosensitivity in subjects with a cervicogenic headache (CGH) remains uncertain. The aim of the study was to evaluate if there was a relationship between the tissue mechanosensitivity and cranio-cervical angle (CCA) that was moderated by pain intensity and/or disability in subjects with CGH. A convenience sample of 102 subjects was recruited. The CCA was measured with photographs, using a postural assessment software. The pain intensity was measured with a visual analogue scale (VAS), and the disability was measured with the Northwick Park Questionnaire. The pressure pain threshold (PPT) was measured at the spinous process of C2, the upper trapezius and splenius capitis muscles, and the median nerve. Simple moderation multiple regression analyses were constructed. There was a positive relationship between PPT at C2 and CCA, but a nonsignificant relationship for the PPT measured at the muscles and median nerve. The effect of PPT at C2 over CCA was moderated by pain intensity (R^2^ = 0.17; R^2^ change = 0.06; *p* < 0.05) but not disability. The Johnson–Neyman analysis revealed a cut-off point for the statistical significance of 4.66 cm in VAS. There seems to be a positive relationship between PPT at C2 and CCA, which is positively moderated by pain intensity in subjects with CGH.

## 1. Introduction

The term cervicogenic headache (CGH) refers to a headache caused by a dysfunction in the cervical spine and its osseous, disc and/or soft tissue components, which is usually accompanied by cervical pain [1] and one-sided pain referred to the head [2]. Cervical pain has been proposed as a possible trigger for headache [3] because of the convergence in the trigeminal nucleus caudalis [4]. Cervical pain is one of the most common musculoskeletal complaints in the adult population, with an annual incidence ranging from 10.4% to 21.3% and a prevalence of up to 86.8% in developed countries [5]. On the other side, headaches have an estimated prevalence of up to 4.6%, with CGH representing 17% of all headache types [6] and generating an annual cost of 173 billion euros in European Union countries [7]. The high prevalence and elevated associated costs explain why the research in this field has increased in the last years [8,9].

It has been suggested that cranio-cervical pain is related to posture alterations [10], forward head posture (FHP) being one of the most investigated, which is characterized by a decreased cranio-cervical angle (CCA) [11] leading to an extension of the occipital and upper cervical spine and a flexion of the medium-lower cervical spine [3]. This posture induces a lengthening of the cervical extensors and occipital flexors muscles, and a shortening of the cervical flexor muscles and suboccipital muscles [12], which are the most shortened [13].

It has been suggested that FHP could impose a great mechanical demand in different tissues in the cranio-cervical region, sensitizing them and thus contributing to the appearance and/or perpetuation of pain [14]. Multiple authors have investigated the relationship between tissue mechanosensitivity, posture alterations and neck pain [15,16,17,18], with some finding an increase of mechanosensitivity in subjects with FHP [17,18]. Furthermore, Castien et al. [16] have found an association between headache intensity and mechanosensitivity for some tissues of the cranio-cervical region [16].

Despite emerging research about the relationship between FHP, tissue mechanosensitivity and cervical pain, it is not known if this relationship exists in subjects with CGH. The aim of the present study is to evaluate if there is a relationship between tissue mechanosensitivity and CCA that is moderated by the pain intensity and/or the degree of disability in subjects with CGH.

## 2. Materials and Methods

### 2.1. Design

A cross-sectional study was conducted following the Strengthening the Reporting of Observational Studies in Epidemiology (STROBE) recommendations [19] and according to the Declaration of Helsinki. Ethical approval was obtained from Universidad CEU Cardenal Herrera (CEI16/011).

### 2.2. Subjects

A convenience sample of subjects with CGH was recruited through announcements in the city of Alcalá de Henares. All subjects signed an informed consent form before participation in the study. Demographic data about the weight, height, age, sex and body mass index (BMI) were collected from all subjects. All subjects met the criteria described in the third edition of the International Classification of Headache Disorders for CGH [1].

The inclusion criteria were: older than 18 years, suffered from cervical pain with associated unilateral headaches from at least three months, and had less than 32 degrees of cervical rotation in the cervical flexion-rotation test evaluated with a cervical goniometer [20]. Subjects were excluded if they presented: systemic diseases, previous cervical surgery, trauma in the last six months, diagnosis of migraine or another possible cause of headaches, and/or neurological symptoms.

### 2.3. Sample Size

The sample size was calculated based on the increase of the determination coefficient (R^2^) with the addition of the interaction term in a hierarchical multiple regression analysis. Assuming that only one predictor (interaction term) was tested with the R^2^ change, nine predictors were included in the model in the first step, there was an effect size of 0.10, 80% power and a value of 0.05, 81 subjects had to be recruited. Assuming a 20% rate of dropouts, the final sample size was composed of 102 subjects.

### 2.4. Measurements

All variables were measured at the University of Alcalá, Physiotherapy and Pain Research Center (Madrid, Spain) by two physiotherapists with more than 10 years of experience in manual therapy who were blinded to each other’s measurements. One physiotherapist evaluated the tissue’s mechanosensitivity, and the other one evaluated the CCA.

#### 2.4.1. Pain Intensity and Disability

A visual analogue scale (VAS) was used for the measurement of the pain intensity during the last week; the VAS is a reliable tool (*r* = 0.94) ranging from 0 (no pain) to 10 (worst imaginable pain) [21]. Disability was measured with the Northwick Park Questionnaire (NPQ), a valid tool with an intraclass correlation coefficient (ICC) value of 0.63, which was transculturally adapted into Spanish (Spain) in 2001 [22]. The NPQ ranges from 0 (no disability) to 100 (maximum degree of disability).

#### 2.4.2. Cranio-Cervical Angle

For the measurement of the CCA, a reflex camera (Nikon Model D5300 SLR, Tokyo Japan) was used to take photographs that were analyzed with a postural assessment software [23,24]. The camera was fixed three meters away from the standing subject, who was told to maintain a relaxed posture. One photograph per subject was taken [23,24].

Two lines were traced using as a reference two markers placed on the tragus of the ear and the spinous apophysis of the 7th cervical vertebrae (C7). The first line was a horizontal line crossing the C7 marker, and the second line was an oblique line linking the C7 and tragus markers. The CCA corresponded to the angle between the two lines. The measurement of the CCA, as stated above, has shown good reliability with an ICC value of 0.98 and good validity in comparison to radiography (*r* = 0.89) [25].

#### 2.4.3. Pressure Pain Threshold

The pressure pain threshold (PPT), measured with a hand-held algometer (Wagner Force Dial, Model FDK 20, Wargner Instruments, Greenwich, CT, USA), was used as an estimator of tissues’ mechanosensitivity. The algometer has a 1-cm^2^ head that records pressure in kg/cm^2^. Participants were told to indicate when the sensation changed from pressure to pain, whilst the evaluator increased the pressure at 1 kg per second. Each location was measured three times, leaving a 30-s rest period between them, and their mean was used for the statistical analysis [26].

The PPT was measured in the upper trapezius and splenius capitis muscles on the painful side. The muscle belly was palpated, with the patient lying in a prone position, to manually locate the most painful point, where the PPT were measured. The PPT measured with a hand-held algometer in cervical muscles has shown good reliability (ICC = 0.87 to 0.89) [27].

The PPT was also measured at the spinous process of the second cervical vertebra (C2), with the patient lying in a prone position. The spinous process of C2 was located just below the occipital bone. Finally, the PPT was measured at the median nerve at the location described by Sterling et al. [28], in the cubital fossa medial to and adjacent to the tendon of the biceps. This procedure has shown good reliability (ICC = 0.92 to 0.97) [28].

### 2.5. Statistical Analysis

For the descriptive analysis of the continuous data, the mean and standard error of the mean as well as the minimum and maximum values were reported. For categorical data, the absolute frequencies and percentages were calculated.

For the analysis of the relationship between PPT and CCA, multiple moderated regression analyses were constructed (PROCESS Model 2). PPT was considered the focal predictor, VAS and NPQ the moderators of the model, and CCA the dependent variable. The age, BMI and sex were included as covariates. The fit of the model was evaluated with the coefficient of determination (R^2^) and the interaction terms with the R^2^ change. If one of the two interactions was statistically significant but the other was not, then a single moderation multiple regression model (PROCESS Model 1) with only the inclusion of the significant interaction was constructed, and the results of the two models were reported. For significant models, the unstandardized regression coefficients (B) with 95% confidence intervals (95% CI), based on a percentile bootstrap with 5000 samples, were calculated [29].

For the interpretation of the interaction terms, the Johnson–Neyman technique for the evaluation of significance intervals was used. The cut-off points of the significance intervals were reported separately for each significant interaction, and a scatterplot of the relationship between the effect of the focal predictor on CCA and the level of the moderator with 95% CI was constructed [29].

All the analyses were conducted using the statistical software SPSS v22.00 (SPSS Inc., Chicago, IL, USA). The regression models were constructed using the PROCESS macro version 3.4 (Andrew F. Hayes^®^) for SPSS [30]. An α level of 0.05 and 95% CI were assumed for all analyses.

## 3. Results

**Figure 1:**
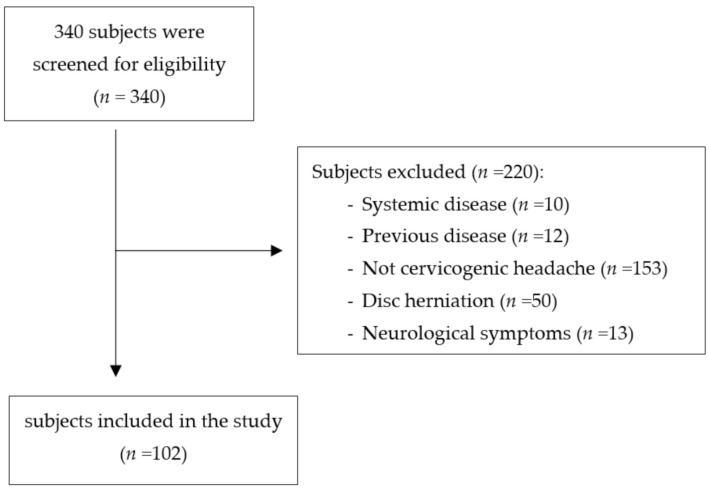
Flow diagram of subjects.

The final sample was composed of 102 subjects (Figure 1) with a mean age of 21.92 ± 0.35 years. The characteristics of the included subjects are presented in Table 1.

### Relationship between Pressure Pain Threshold and Cranio-Cervical Angle

The multiple moderation regression analyses for the prediction of CCA were nonsignificant for the focal predictors that PPT measured at the upper trapezius muscle (R^2^ = 0.04, *p* = 0.84), splenius capitis muscle (R^2^ = 0.06, *p* = 0.72) and median nerve (R^2^ = 0.03, *p* = 0.92).

The multiple moderation regression analysis for the prediction of CCA by the focal predictor that PPT measured at C2 revealed a significant interaction between PPT at C2 and pain intensity (R^2^ = 0.19; R^2^ change = 0.07; *p < 0.05*). The model unstandardized regression coefficients are presented in Table 2.

The results of the single moderation multiple regression analysis (R^2^ = 0.17; R^2^ change = 0.06; *p <* 0.05) conducted after the exclusion of the interaction term between the pressure pain threshold at C2 and NPQ are presented in Table 3.

**Figure 2:**
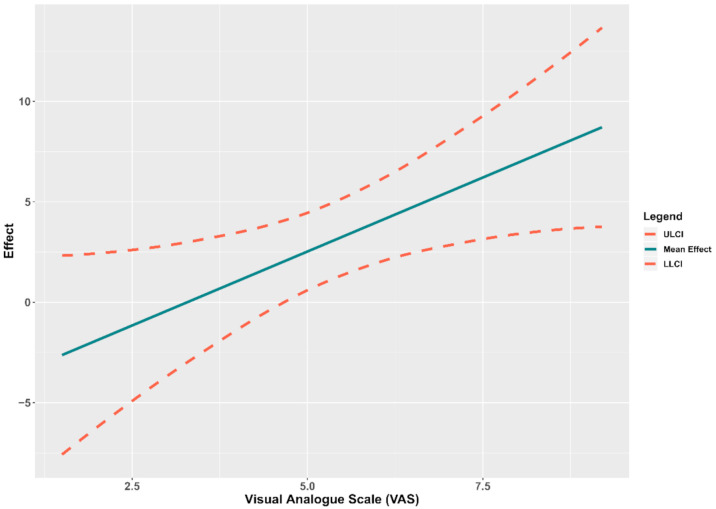
Conditional effect of the pressure pain threshold measured at C2 over the cranio-cervical angle depending on the level of the visual analogue scale. Abbreviations: ULCI, upper limit of the 95% confidence interval; LLCI, lower limit of the 95% confidence interval.

The Johnson–Neyman analysis of the region of significance for the single moderation multiple regression analysis revealed a cut-off point for the statistical significance of 4.66 cm in VAS (43.56% below and 56.44% above). There was a significant relationship between the PPT measured at C2 and CCA above the threshold of 4.66 cm in VAS (Figure 2).

## 4. Discussion

The aim of the present study was to evaluate if the relationship between the tissue mechanosensitivity and CCA was moderated by pain intensity and/or disability in subjects with CGH. The main result was the presence of a relationship between PPT measured at C2 and CCA, which was moderated by pain intensity (B = 1.52; CI 95%, 0.22 a 2.95).

### 4.1. Relationship between Pressure Pain Threshold and Cranio-Cervical Angle

It has been observed that FHP produces an increase in mechanical stress in some cranio-cervical structures [14] The mechanical over-demands have been related to an increase in mechanosensitivity measured with PPT [17]. In the present study, there was a significant positive relationship between the PPT measured at C2 and CCA, i.e., subjects with a decrease in CCA (increased FHP) had less PPT at C2 (increased tissue mechanosensitivity). It has been proposed that FHP could increase the neural mechanosensitivity of the C2 nerve root through a sustained mechanical stress induced by the shortening of the suboccipital muscles [12,31]. Furthermore, the increase in the sustained pressure over the articular surfaces of the upper cervical vertebras because of FHP could lead to a peripherical and/or central sensitization, which in turn would increase the neural mechanosensitivity, thus playing an important role in the pathophysiology of CGH [6,32]. Regarding this topic, Martinez–Merinero et al. [17] and Amiri et al. [33] found a decrease in PPT measured over the articular process of C2 in subjects with FHP [17] and CGH [33].

On the other hand, there was a nonsignificant relationship between the PPT measured at the upper trapezius and splenius capitis muscles, and CCA. These results agree with the ones obtained by Martinez–Merinero et al. [17] and Kocur et al. [34], who did not find an increase in the mechanosensitivity of the upper trapezius and sternocleidomastoid muscles in subjects with FHP. However, Pacheco et al. [18] and Huber et al. [35] found an increase in the mechanosensitivity of the upper trapezius muscle in subjects with FHP. Furthermore, Martinez–Merinero et al. [17] found an increase in the mechanosensitivity of the splenius capitis muscle. In 1992, Bovim [36] found a decrease in the PPT measured in multiple points in the cranio-cervical region in subjects with CGH when compared to healthy subjects, subjects with migraine and subjects with tension-type headaches; however, they did not measure any point evaluated in the present study [36]. These discrepancies can result from the different procedures used for the measurement of PPT in the different muscles, such as the point of measurement within the same muscle over different studies [17,34,36,37,38]. Furthermore, FHP inducing a great mechanical stress over the suboccipital muscles but no other cranio-cervical muscles [13,31] could also explain the observed discrepancies, due to the mechanical stress adaptation capability of these muscles over different subjects.

Finally, there was a nonsignificant association between the PPT measured at the median nerve and CCA. This result disagrees with Martinez–Merinero et al. [17], who found an increase in the mechanosensitivity of the median, radial and ulnar nerves in subjects with FHP. These discrepancies could be because Martinez–Merinero et al. [17] mainly measured subjects without pain, so that the presence of pain could modify the relationship between mechanosensitivity and CCA. However, the results of this study agree with the ones obtained by other researchers who have measured the mechanosensitivity of the upper limb nerves with neural tension tests in subjects with CGH [37] and in subjects with neck pain [39]. The discrepancies can be due to the fact that although FHP induces a sustained stress over some cranio-cervical structures, the median nerve depends mostly on the brachial plexus emerging from C5-T1. Furthermore, Julius et al. [40] found that FHP did not induce a movement over the median nerve at the forearm.

### 4.2. Interaction between Mechanosensitivity at C2, Pain Intensity and Disability

It was found that the pain intensity moderated the effect of C2 over CCA, but there was a nonsignificant moderation effect for disability that was measured with NPQ.

Some authors have proposed the concept of tissue irritability and have related this concept to high pain intensity levels, so that the higher the pain intensity, the higher the tissue irritability [41]. The increase in tissue irritability decreases the mechanical stress tolerance capability [41,42]; thus, the concept of irritability, which can be indirectly measured with pain intensity and/or disability, should be taken into account when evaluating the relationship between posture alterations and tissue mechanosensitivity. In the present study, it was found that the positive relationship between PPT at C2 and CCA increased as the pain intensity increased, i.e., subjects with a higher irritability tend to have a lower stress adaptation capability, and thus posture alterations that increase mechanical stress are more related to tissues’ mechanosensitivity. However, the effect size of this interaction was small (R^2^ change = 0.06), which means that the interaction term only accounted for an additional 6% of the variability of CCA; thus, the results of the present study should be interpreted with caution.

Subjects with neck pain tend to have high levels of disability [43]. Neck disability has been associated with neck pain intensity [44] and FHP [10,44,45]. This relationship has also been observed in mid-term-aged [46] and elderly [47] subjects with CGH. In the present study, it was found that the relationship between PPT measured at C2 and CCA was not moderated by neck-related disability. Many factors influence the perception of disability; thus, although it could be suggested that a subject with high irritability levels would have a high disability, it is not as plausible that a subject with a high disability would have a high irritability. Based on the results of the present study, it seems necessary for future research relating to the relationship between posture, pain and mechanosensitivity to take into account the possible interaction between these variables, to improve the statistical power of the studies and the reproducibility of their results.

### 4.3. Limitations

Our study has several limitations that must be considered. First, the age of our sample is very limited (18–33 years old), with a higher percentage of women, reducing the external validity of our results. Second, the significant association that was found between the PPT measured at C2 and CCA should be interpreted with caution, as it accounted for 17% of the variability of CCA. Finally, future research with other predictor and moderator variables is needed.

## 5. Conclusions

There seems to be a positive relationship between PPT measured at C2 and CCA that is positively moderated by pain intensity in subjects with CGH, so that the higher the pain intensity, the higher the relationship between the PPT at C2 and CCA. However, there seems to be no moderation effect of neck-related disability. Future research should consider moderation effects when evaluating the relationship between posture, pain and tissue mechanosensitivity.

## Figures and Tables

**Table 1:** Characteristics of the subjects (*n* = 102).

Variable	Mean ± SE (Minimum–Maximum)
Age, years	21.92 ± 0.35 (18.00–33.00)
Height, cm	169.18 ± 0.97 (153.00–196.00)
Weight, kg	62.60 ± 1.21 (43.00–106.40)
BMI, kg/m^2^	21.65 ± 0.26 (15.70–32.12)
Sex, *n* (%)	
Women	73 (72.3)
Men	28 (27.7)
CCA, degrees	50.29 ± 0.52 (38.00–63.00)
VAS, cm	5.03 ± 0.16 (1.50–9.20)
NPQ	16.59 ± 0.78 (2.77–38.00)
PPT, kg/cm^2^	
Upper trapezius	2.47 ± 0.07 (1.40–4.18)
Splenius capitis	2.07 ± 0.05 (1.15–4.15)
Median nerve	1.66 ± 0.04 (1.00–2.95)
C2	2.20 ± 0.05 (1.20–3.60)

Abbreviations: SE, standard error of the mean; BMI, body mass index; CCA, cranio-cervical angle; VAS, visual analogue scale; NPQ, Northwick Park Questionnaire; PPT, pressure pain threshold; C2, spinous process of the second cervical vertebra.

**Table 2:** Multiple moderation regression analysis for the prediction of the cranio-cervical angle with the pressure pain threshold measured at C2 as the focal predictor.

Variable	B * (SE)	95% CI
Age	0.11 (0.15)	−0.19 to 0.41
BMI	0.07 (0.17)	−0.28 to 0.40
Sex	0.57 (1.33)	−2.04 to 3.16
PPT C2	−3.38 (4.06)	−11.51 to 4.44
VAS	−4.38 ^†^ (1.76)	−7.87 to −1.02
NPQ	0.44 (0.26)	−0.04 to 1.00
Interaction 1	1.79 ^†^ (0.70)	0.45 to 3.21
Interaction 2	−0.18 (0.11)	−0.42 to 0.02

* Unstandardized regression coefficient; ^†^ Statistically significant (*p* < 0.05); Interaction 1 refers to the interaction between PPT at C2 and VAS; Interaction 2 refers to the interaction between PPT at C2 and NPQ. Abbreviations: SE, standard error; CI, confidence interval; BMI, body mass index; PPT, pressure pain threshold; VAS, visual analogue scale; NPQ, Northwick Park Questionnaire.

**Table 3:** Single moderation multiple regression analysis for the prediction of the cranio-cervical angle with the pressure pain threshold measured at C2 as the focal predictor.

Variable	B * (SE)	95% CI
Age	0.10 (0.16)	−0.22 to 0.40
BMI	0.09 (0.17)	−0.26 to 0.43
Sex	0.77 (1.32)	−1.82 to 3.33
PPT C2	−4.99 (3.91)	−12.62 to 2.68
VAS	−3.70 ^†^ (1.76)	−7.33 to −0.43
NPQ	0.03 (0.06)	−0.09 to 0.16
Interaction	1.52 ^†^ (0.70)	0.22 to 2.95

* Unstandardized regression coefficient; ^†^ Statistically significant (*p* < 0.05); Interaction term refers to the interaction between PPT at C2 and VAS. Abbreviations: SE, standard error; CI, confidence interval; BMI, body mass index; PPT, pressure pain threshold; VAS, visual analogue scale; NPQ, Northwick Park Questionnaire.

## Data Availability

The data presented in this study are available on request form the corresponding author. The data are not publicly available due to privacy issues.
